# Reliable handling of highly A/T-rich genomic DNA for efficient generation of knockin strains of *Dictyostelium discoideum*

**DOI:** 10.1186/s12896-016-0267-8

**Published:** 2016-04-14

**Authors:** Asuka Mukai, Aya Ichiraku, Kazuki Horikawa

**Affiliations:** Division of Bioimaging, Institute of Biomedical Sciences, Tokushima University Graduate School, 3-18-15 Kuramoto-cho, Tokushima City, Tokushima 770-8503 Japan

**Keywords:** A/T-rich genome, Linear DNA cloning, Knockin, *Dictyostelium discoideum*

## Abstract

**Background:**

Social amoeba, *Dictyostelium discoideum*, is a well-established model organism for studying cellular physiology and developmental pattern formation. Its haploid genome facilitates functional analysis of genes by a single round of mutagenesis including targeted disruption. Although the efficient generation of knockout strains based on an intrinsically high homologous recombination rate has been demonstrated, successful reports for knockin strains have been limited. As social amoeba has an exceptionally high adenine and thymine (A/T)-content, conventional plasmid-based vector construction has been constrained due to deleterious deletion in *E. coli*.

**Results:**

We describe here a simple and efficient strategy to construct *GFP*-knockin cassettes by using a linear DNA cloning vector derived from N15 bacteriophage. This allows reliable handling of DNA fragments whose A/T-content may be as high as 85 %, and which cannot be cloned into a circular plasmid. By optimizing the length of recombination arms, we successfully generate *GFP*-knockin strains for five genes involved in cAMP signalling, including a triple-colour knockin strain.

**Conclusions:**

This robust strategy would be useful in handling DNA fragments with biased A/T-contents such as the genome of lower organisms and the promoter/terminator regions of higher organisms.

**Electronic supplementary material:**

The online version of this article (doi:10.1186/s12896-016-0267-8) contains supplementary material, which is available to authorized users.

## Background

The eukaryote *Dictyostelium discoideum* (*D. discoideum*), also called social amoeba, is an excellent model to study the principles of unicellular physiology and multicellular development. In addition to the small genome size (34 Mb encoding ~13,500 genes) [1], its haploidy is highly compatible with the functional analysis of genes by mutational approaches. Genome-wide mutations have been randomly introduced by chemical mutagenesis and semi-randomly by restriction enzyme mediated integration mutagenesis (REMI) [2, 3]. Homologous recombination has also been effective for introducing site-specific mutations, or for the targeted disruption of genes of interest (GOI). As with other model organisms, gene targeting has been performed by introducing a DNA cassette consisting of a selection marker flanked by 5′ and 3′ recombination arms. For *D. discoideum*, up to a few kb of homology arm was sufficient to obtain knockout strains with high efficiency (>20 %) [4, 5].

Knockin, while also utilizing homologous recombination, would be a powerful strategy. 5′- or 3′-terminal tagging of genes with fluorescent proteins (FPs) at the endogenous locus is highly useful for quantitative analysis of protein abundance and cellular localization [6, 7]. Replacing the endogenous promoter with a synthetic one such as the tetracycline-inducible system (Tet-system) is another application which allows efficient control of the gene expression level [8–10]. In spite of potential applicability, successful generation of knockin strains has been limited to a few loci, while knockout strains have been routinely generated [6–12].

One possible explanation for this constraint is the technical difficulty in constructing knockin DNA cassettes compared with knockout DNA cassettes. The DNA cassette for gene tagging and promoter replacement must harbour terminator or promoter sequences from the endogenous targeted gene. The genome of *D. discoideum* has an intrinsically high A/T-content, with the promoter, terminator and intron sequences often showing >85 % A/T-content while that of exons is more moderate (~70 % of A/T) [1]. As has been widely accepted, A/T-rich or repetitive DNA fragments are notoriously unstable in circular plasmids due to secondary structures and the functional interference with transcription and replication systems of the host *E. coli* [13–16].

Cloning of unstable DNA has been enhanced by the use of modified plasmid vectors and host *E. coli* [14]*.* Low-copy number and transcription-free plasmids have been utilized for cloning small A/T-rich fragments. Recombinase-deficient *E. coli* strains have been developed to increase the stability of cloned DNAs containing repetitive sequences. However, construction of tagging vectors by using a circular plasmid was unsuccessful in our trial. We routinely experienced difficulties in the cloning of promoter or 3′ UTR/terminator sequences whose A/T-contents exceeded 75 %. Once a knockin cassette was successfully constructed, we often experienced serious instability during its maintenance in *E. coli* even when we utilized the improved materials described above.

To circumvent these issues, we employed a recently developed linear DNA cloning system that allows robust handling of large and A/T-rich DNA fragments [17–19]. We first demonstrated the efficient construction of 3′-tagging vectors by a simple restriction-ligation method. We then optimized the size of recombination arms for efficient knockin, and identified that the critical arm length differs depending on the target locus. Robustness of our strategy was finally demonstrated by multiple labelling of a gene with three different fluorescent proteins. These results suggest that our simple strategy would facilitate genomic manipulations which had previously been hampered by the inability to clone DNA fragments with biased A/T-contents for a variety of model organisms.

## Methods

### General molecular biology

For the preparation of genomic DNA, amoeba cells were suspended in quick lysis solution (50 mM KCl, 10 mM Tris pH 8.3, 2.5 mM MgCl_2_, 0.45 % NP40, 0.45 % Tween 20 and 1 μg/μl of proteinase K) to ~50 cells/μl. Cells were lysed at 55 °C for 10 min, then heat denatured at 95 °C for 5 min. 1 μl of cell lysate was analysed by PCR. PCR for DNA cloning and genome analysis was performed by using KODplus (TOYOBO) and EmeraldAmp (TAKARA), respectively. PCR was performed according to the touch-down protocol as follows: (94°C_1 min, 60 °C_30 s, 60 °C_1 min/kb) × 5 cycles; followed by (94°C_1 min, 53°C_30 s, 60 °C_1 min/kb) × 5 cycles; (94 °C_1 min, 46°C_30 s, 60 °C_1 min/kb) × 5 cycles; and (94°C_1 min, 40°C_30 s, 60 °C_1 min/kb) × 25 cycles. Transcripts from knockin loci were RT-PCR amplified using an oligo-dT primer. Sequence verified cDNAs were subcloned into *pDM304*, the extra-chromosomal plasmid vector for *D. discoideum*, to establish cells over-expressing tagged proteins [20]. Southern blotting was performed as described in Additional file 1.

### Handling A/T-rich DNA

Extension of PCR was carried out at 60 °C as A/T-rich fragments are often poorly amplified at higher temperatures [21]. DNA fragments separated by gel electrophoresis were stained with Gel-Red (Biotium) then were visualized by a blue light illuminator instead of UV to minimise photo-induced DNA damage. DNA fragments were collected by mechanical filtration by using a GenElute Agarose spin column kit (SigmaAldrich: G2291-70EA). Note that the chemical elution kit was not appropriate as A/T-rich DNAs are irreversibly denatured in the presence of chaotropic agents such as guanidinium and iodide ions [22].

### Universal knockin module

To create universal knockin modules encoding fusion-ready fluorescent proteins followed by the excisable selection marker (Additional file 2: Figure S1), three amplicon units were sequentially cloned between the BamHI and ApaI site of pBluescript SK. Unit 1: cDNA of FPs sandwiched between a fusion linker (encoding 26 amino acids, Additional file 2: Figure S1) and the 1st LoxP site (BamHI > HindIII). Unit 2: Terminator of *myosin heavy chain A* (HindIII > SalI). Unit 3: *BsR*-selection marker with the 2nd LoxP site amplified from pLPBLP [23] (SalI > ApaI). To allow efficient expression of fluorescent proteins in *D.d* cells, cDNA from *Aequorea victoria* (*a.v.*) was utilized. To generate *a.v._mEGFP*, F64L, S65T, Y100F, S108T, M141L, A206K and I219V mutations were introduced into the original *GFP* by site directed mutagenesis. To generate *a.v._Turquoise2* [24], F64L, Y66W, S72A, Y100F, S108T, M141L, N146F, H148D, M153T, V163A, S175G, I219V and H231L were introduced to the original GFP. *mRFPmars* whose codon usage was optimised for *D. discoideum*, was PCR amplified from *pmRFPmars-LimΔcoil* (Dicty Stock Center ID_475) [25]. Resulting plasmids, *pUniv_CKI_mEGFP*, *_Turq2* and *_mRFPmars* whose GenBank Accession numbers are KU163138, KU163139 and KU163140, respectively, were deposited in the Dicty stock center.

### Construction of knockin vectors by using linear cloning system

DNA fragments for the knockin vector were assembled by using the pJAZZ linear DNA cloning system (Lucigen, #43036 and 43042). We first PCR amplified the 3′ recombination arm (i.e., 3′ UTR/terminator) and cloned it into the pJAZZ-OK_blunt vector according to the manufacturer’s instructions. 10–30 μg of pJAZZ vector harbouring the 3′ recombination arm in the correct orientation was digested with ApaI and the resulting shorter fragment was separated by gel electrophoresis. The fragment was filter-eluted and concentrated to >100 ng/μl after desalting by ethanol precipitation. Similarly, a long arm of NotI digested pJAZZ control vector, NotI/BamHI digested 5′ recombination arm of GOI and BamHI/ApaI digested knockin module were prepared. 100 ng each of these four fragments were directionally ligated by single tube reaction and electroporated into TSA *E. coli.* Colonies were isolated from a YT-agar plate containing 30 μg/ml of kanamycin and screened by PCR and restriction enzyme digestion. For a large-scale preparation of the knockin vector, transformed TSA *E. coli* were cultured in 100 ml of TB medium. Approximately 100 μg of knockin vector was routinely obtained using the NucleoBond Xtra Midi kit (MACHEREY-NAGEL). To prepare the transformation-ready knockin cassette, 100 μg of knockin vectors were digested with NotI for 10 h then were cleaned-up by phenol-chloroform extraction and ethanol precipitation without DNA-size fractionation. The knockin DNA cassettes were suspended in sterile water (2 μg/μl) and were stored at -30 °C until use.

### Cell culture

Axenic strain Ax2 was cultured and transformed as described elsewhere [4, 23, 26]. For knockin transformation, cells were washed and suspended with ice-cold EP buffer (6.6 mM KH_2_PO_4_, 2.8 mM Na_2_HPO_4_, 50 mM sucrose, pH 6.4) to 1 × 10^7^ cells/ml. 800 μl of cell suspension mixed with 10–40 μg of NotI digested knockin vector in a 4-mm width cuvette was subjected to electroporation (5 s separated two pluses with 1.0 kV and 1.0 ms of the time constant) by using a MicroPulser (Bio-Rad). These cells were plated on 4–6 pieces of 10 cm-petri dishes with HL5 medium and incubated at 22 °C for 18 h under the non-selective condition, then were cultured in the presence of 12.5 μg/ml Blasticidin S (Wako). After 4–7 days, colonies of candidate recombinants were manually picked and were transferred into 96-well plates, then subjected for the genome check by PCR and Southern blotting analysis. Cre-recombinase mediated removal of the selection cassette was performed as described previously [23]. Briefly, NLS-Cre was transiently expressed by introducing *pDEX_NLS-Cre*. Candidate clones were selected in the presence of 5 μg/ml of G418 for 2–5 days followed by an additional few days’ culture in the absence of G418 (LifeTechnologies). Optionally, single cell sorting of isolated clones was performed by a JSAN cell sorter equipped with a CloneMate module (Bay Bioscience).

### Immunoblotting

Cells starved for 8 h were lysed with SDS sample buffer. Proteins (3.6 × 10^5^ cells/lane) were separated by SDS-polyacrylamide gel electrophoresis (5–15 % gradient gel, BIO CRAFT) and blotted on the PVDF membrane. *GFP/Turquoise2*- or *mRFPmars*-tagged proteins were detected with a rabbit monoclonal antibody to *GFP* (Abcam, ab183735) and a rat monoclonal antibody to *RFP* (ChromoTek, 5 F8), respectively.

### Imaging

Triple-colour knockin cells on a 10-cm petri dish (8 × 10^6^ cells/dish) were starved for 8–10 h in Development Buffer (5 mM Na_2_HPO_4_, 5 mM KH_2_PO_4_, 1 mM CaCl_2_, 2 mM MgCl_2_). These cells were re-plated on a 35 mm-glass bottomed dish (Iwaki) and were left at 22 °C for 30 min allowing them to establish the aggregation stream. Live cell images were captured on an inverted confocal microscope (Nikon A1R, Nikon) equipped with PlanApoVC 60xWI (NA 1.2), multi Argon gas laser (457, 488 nm, Melles Griot) and 561 nm DPSS laser (Melles Griot).

## Results

### Construction of A/T-rich knockin vector by a linear cloning system

**Fig. 1:**
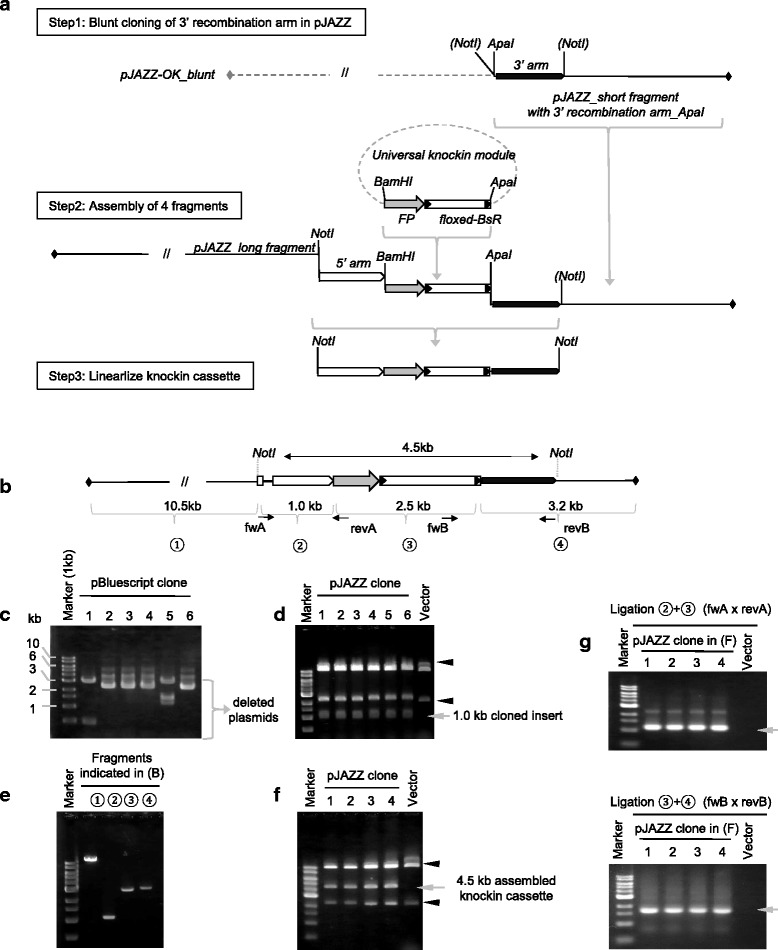
Knockin vector construction by a linear DNA cloning system. **a** 3-step construction of the 3′-tagging vector. Step 1: Preparation of pJAZZ vector harbouring A/T-rich 3′ recombination. Step 2: Assembly of the knockin vector by 4-piece ligation. Step 3: Release of knockin cassette by NotI digestion. **b** Design of *GFP* knockin vector for DDB_G0273397/*carA-1* harbouring 1.0 kb each of 5′ and 3′ recombination arms. **c**, **d** Stable cloning of A/T-rich 3′ UTR/terminator of *carA-1* by linear cloning system. 1 kb of 3′ UTR/terminator of *carA-1* were blunt cloned into pBluescript (**c**) and pJAZZ vector (**d**). Release of the insert in randomly selected 6 DNA clones was checked by restriction enzyme digestion with XhoI and SpeI for pBluescript and with NotI for pJAZZ (these are the multiple cloning sites on each vector). Variable size of released fragments in C indicates deletions of circular plasmids. Appropriate size of inserts (*arrow* in **d**) were released from all the 6 clones of pJAZZ vector. The lane for negative control (Vector) was loaded with NotI-digested pJAZZ vector carrying no insert. Arrow heads represent the long and short arm of NotI-digested pJAZZ vector. **e** Four DNA fragments as depicted in B were subjected to directional ligation. **f** DNAs from randomly selected TSA *E. coli* clones were digested with NotI to excise the 4.5 kb of assembled knockin cassette (*arrow*). **g** Appropriate DNA assembly in 4 clones (same as in **f**) was detected by PCR for fragment ligation between 2 and 3 (*upper column*, 2 + 3) or fragment 3 and 4 (*bottom column*, 3 + 4). Primer position was depicted in (**b**). All the molecular marker was1 kb DNA ladder

To construct >4 kb of A/T-rich *GFP* knockin cassette consisting of 5′ and 3′ recombination arms (~1.0 kb each) centred by the knockin module encoding the fusion ready *GFP* and the selection marker (*BsR*; blasticidin resistance), we designed a simple experimental scheme that utilizes a linear DNA cloning system which allows robust handling of large and A/T-rich DNA (Fig. 1a). To facilitate vector construction, a universal knockin module was independently prepared as *pUniv_CKI_mEGFP* (Fig. 1a and Additional file 2: Figure S1). In brief, it contains cDNA encoding *mEGFP* followed by a generic terminator from *myosin heavy chain A* (*MHC*), which allows the expression of a *GFP*-fusion protein just after the knockin event. Two LoxP sites were introduced for Cre-mediated excision of the *MHC* terminator and *BsR* expression unit, which is needed for recycling the *BsR* marker and for restoring appropriate genomic organization to allow normal gene expression under the control of the endogenous 3′ UTR/terminator of the GOI.

To validate this set-up, we created a *GFP-*knockin cassette for *carA-1*(DDB_G0273397), encoding cAMP receptor during aggregation (Fig. 1b). We cloned PCR amplified 3′ UTR/terminator (1.0 kb) of *carA-1* into pJAZZ-OK_blunt vector [17]. Although it was not possible to clone this 1.0 kb fragment with 85 % A/T content into a circular plasmid due to the occurrence of deletions (Fig. 1c), pJAZZ vector allowed robust cloning without any signs of deletions and rearrangements (Fig. 1d) which was separately confirmed by nucleotide sequencing (data not shown). The unique ApaI site at the cloning site was utilized to prepare the shorter arm of pJAZZ vector harbouring a 3′ recombination arm of *carA-1* and the kanamyicin resistance unit. Other DNA fragments corresponding to the longer arm of the pJAZZ vector (a NotI-digested 10.5 kb fragment including the replication origin), PCR amplified and NotI/BamHI digested 5′ recombination arm (72 % A/T), and BamHI/ApaI digested universal knockin module (72 % of A/T) were similarly prepared (Fig. 1e and Table 1). These four DNA fragments were directionally ligated in vitro and were transformed into TSA competent cells, which is the optimized host *E. coli* for pJAZZ vector [17]. Assembly of these fragments was efficient as 75 % of checked clones were identified as carrying an appropriately ligated 4.5 kb knockin cassette for the *carA-1* gene (Fig. 1f-g). We also successfully constructed knockin vectors for other genes involved in cAMP signalling, containing a range of different recombination arm lengths (100–50 % efficiency, Table 1), demonstrating the robustness and general applicability of this strategy to construct the large and A/T-rich knockin DNA cassette.

**Table 1:** Efficiency of knockin vector construction by using a linear DNA cloning system

		Arm length		A/T-content				
Gene	Vector name	5′ arn	3′ arm	5′ arm	3′ arm	Knockin cassette Total	Positive clone	Efficiency
*carA-1* DDB_G0273397	pJ_carA1_GFP	1.0 kb	1.0 kb	72 %	85 %	75 % (4.6 kb)	12/16	75 %
*acaA* DDB_G0281545	pJ_acaA_GFP	0.6 kb	0.5 kb	74 %	83 %	72 % (3.7 kb)	8/8	100 %
*regA* DDB_G0284331	pJ_regA_GFP_0.3	0.3 kb	1.0 kb	69 %	79 %	73 % (3.8 kb)	8/8	100 %
	pJ_regA_GFP_0.5	0.5 kb	1.0 kb	69 %	79 %	73 % (4.0 kb)	7/8	88 %
	pJ_regA_GFP_1.3	1.3 kb	1.0 kb	76 %	79 %	74 % (4.8 kb)	7/8	88 %
dagA/*crac* DDB_G0285161	pJ_crac_GFP_0.3	0.3 kb	0.6 kb	69 %	80 %	72 % (3.7 kb)	8/8	100 %
	pJ_crac_GFP_1.9	1.9 kb	0.6 kb	70 %	80 %	72 % (5.0 kb)	11/16	69 %
*erkB* DDB_G0283903	pJ_erkB_GFP_0.5/0.6	0.5 kb	0.6 kb	64 %	66 %	70 % (3.6 kb)	8/8	100 %
	pJ_erkB_GFP_1.0/0.6	1.0 kb	0.6 kb	72 %	66 %	71 % (4.1 kb)	7/8	88 %
	pJ_erkB_GFP_1.0/1.7	1.0 kb	1.7 kb	72 %	77 %	74 % (5.2 kb)	4/8	50 %
	pJ_erkB_GFP_0.5/1.7	0.5 kb	1.7 kb	64 %	77 %	73 % (4.7 kb)	4/8	50 %

### Optimized conditions for *GFP* knockin

**Fig. 2:**
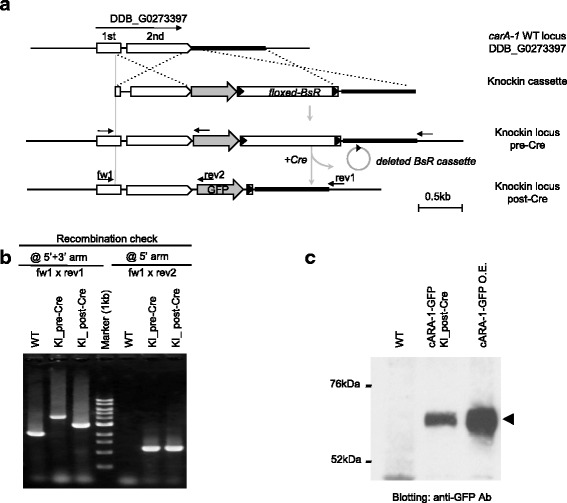
Generation of *GFP* knockin strain for *carA-1.* **a** Genomic organization of wild-type (WT) and *GFP* knockin locus for DDB_G0273397/*carA-1.* **b** WT and knockin locus before and after the removal of *BsR* cassette was detected by PCR. The primer set fw1/rev1, both located outside the homology arms, detects WT and knockin locus (pre_Cre) as 2.0 and 4.5 kb, respectively. *BsR*-removal was detected as the decrease in the size of target locus from 4.5 kb (pre-Cre) to 2.7 kb (post-Cre). Primer combination of rev2 for *GFP* and fw1 confirms specific recombination at the 5′ arm by yielding a 1.0 kb fragment. **c** Expression of *GFP*-tagged *cARA-1* protein of the knockin strain detected by western blotting. Lysate of cells over-expressing *cARA-1-GFP* from extrachromosomal plasmid were loaded as the detection control (1/10 volume, *cARA-1-GFP* O.E)

The amount of targeting cassette and the length of recombination arms have been shown to control the homologous recombination rate. To determine the minimum amount of DNA vector for 3′-terminal knockin, 10, 20 or 40 μg of NotI-digested knockin vector for *carA-1* was electroporated into wildtype cells (Ax2). While none or few blasticidin resistant (*BsR*) colonies were obtained from cells transformed with 10 and 20 μg of linearized vector, more than 500 candidate clones grew under the selective culture conditions when 40 μg of total DNA was introduced. PCR analysis specifically detecting the homologous recombination event at both the 5′ and 3′ arms identified that 28 % of cells transformed with 40 μg of vector were positive clones whose *carA-1* locus was replaced by the knockin cassette (Fig. 2a-b and Table 2). Southern blotting (Additional file 3: Figure S2), genomic sequencing (data not shown) and immunoblotting analysis (Fig. 2c) confirmed the specific expression of *cARA-1-GFP* protein that is not the result of random integration of knockin vector. We concluded that 40 μg of total DNA, which corresponds to ~10 μg of knockin cassette, is optimal to obtain knockin cells.

**Table 2:** Effect of the arm length variation on knockin efficiency

Gene	Chromosome	Vector name	5′ arm length	3′ arm length	Total amount of knockin vector	^a^BsR clone	^b^Positive clone	Efficiency
*carA-1* DDB_G0273397	2	pJ_cAR1_GFP	1.0 kb	1.0 kb	10 μg	0	-	0 %
					20 μg	3	0/3	0 %
							0/0	-
					40 μg	>500	27/96	28 %
							3/16	19 %
*acaA* DDB_G0281545	3	pJ_acaA_GFP	0.6 kb	0.5 kb	40 μg	>200	5/11	46 %
							3/8	38 %
*regA* DDB_G0284331	4	pJ_regA_GFP_0.3	0.3 kb	1.0 kb	40 μg	124	0/96	0 %
							0/28	0 %
		pJ_regA_GFP_0.5	0.5 kb	1.0 kb	40 μg	138	0/96	0 %
							0/48	0 %
		pJ_regA_GFP_1.3	1.3 kb	1.0 kb	40 μg	180	56/96	58 %
							9/16	56 %
dagA/*crac* DDB_G0285161	4	pJ_crac_GFP_0.3	0.3 kb	0.6 kb	40 μg	>500	0/96	0 %
							0/96	0 %
		pJ_crac_GFP_1.9	1.9 kb	0.6 kb	40 μg	>500	10/16	63 %
							8/51	16 %
*erkB* DDB_G0283903	4	pJ_erkB_GFP_0.5/0.6	0.5 kb	0.6 kb	40 μg	>500	0/96	0 %
							0/96	0 %
		pJ_erkB_GFP_1.0/0.6	1.0 kb	0.6 kb	40 μg	>500	0/288^c^	0 %
							0/96	0 %
		pJ_erkB_GFP_1.0/1.7	1.0 kb	1.7 kb	40 μg	>500	16/30	53 %
							4/16	25 %
		pJ_erkB_GFP_0.5/1.7	0.5 kb	1.7 kb	40 μg	>500	14/30	47 %
							5/16	31 %

^a^Total number of *BsR* clones from two experiments by using independently prepared materials

^b^The number of homologous recombinants obtained from two independent experiments was separately provided

^c^One clone with single cross-over at 5′ arm obtained from 288 of *BsR* colonies

We next investigated how the length of the recombination arm affects knockin efficiency. It has been demonstrated that the minimum length of homology arms differs significantly among eukaryotic species. For knockout, as short a length as 20 nucleotides is sufficient for budding yeast, *S. cerevisiae* [27]. Much longer arms up to 10 kb are needed for mice and malaria parasites [18, 28], while less than a few kb are sufficient for *D. discoideum* [23]. By using knockin vectors with distinct lengths of recombination arms, we tried to generate knockin strains for an additional four genes involved in cAMP signalling, as the recombination efficacy was expected to differ depending on the genomic locus. When cells were transformed with a knockin vector harbouring short recombination arms (0.5 kb each for 5′ and 3′) for the *acaA* gene, encoding adenylate cyclase during aggregation, appropriate recombinants were efficiently obtained (46 %), suggesting that homology arms as short as a few hundred base-pairs would be sufficient. For the *regA* gene, encoding cAMP specific intracellular phosphodiesterase, three constructs with 0.3, 0.5 and 1.3 kb of 5′ arm in combination with 1.0 kb of 3′ arm were examined. Although the vector with 0.3 or 0.5 kb of 5′ arm yielded no recombinants, that with 1.3 kb of 5′ arm yielded 58 % of positive colonies, indicating that a longer 5′ arm is needed for *regA* locus. This is also the case for the *crac* gene (DDB_G0285161); encoding cytoplasmic regulator of *acaA*, such that 1.9 kb, but not 0.3 kb of 5′ recombination arm was needed for successful knockin. In the case of MAP kinase gene, *erkB*, a longer 3′ arm rather than 5′ arm was required. Vectors with 0.5 kb of each of homology arm yielded no recombinants from 96 screened colonies. Extending the 5′ arm to 1.0 kb was not effective, although one clone with a single cross-over at the 5′ arm (i.e., integration) was obtained from 288 *BsR*-clones. We then tested a knockin cassette whose 3′ arm was extended to 1.7 kb, reaching into the 2nd exon of the inversely located neighbouring gene, *pigA* (DDB_G0283965*),* while the 5′ arm was kept short (0.5 kb). In this case, 48 % of screened clones were identified to be knockin cells (14/30). The efficiency was comparable to the targeting vector harbouring long homology arms both for 5′ and 3′ arms (1.0 and 1.7 kb, respectively, 53 %). Although we did not examine much longer recombination arm, these results suggest that the efficiency of homologous recombination in *D. discoideum* was not linearly enhanced by longer arm, rather it was simply controlled in all-or-none fashion (Table 2). All together, these results demonstrate that the length of recombination arm up to 3 kb would be sufficient for 3′-tagging, in which downsizing was possible depending on the genomic loci.

### Multicolour labelling by iterative knockin

**Fig. 3:**
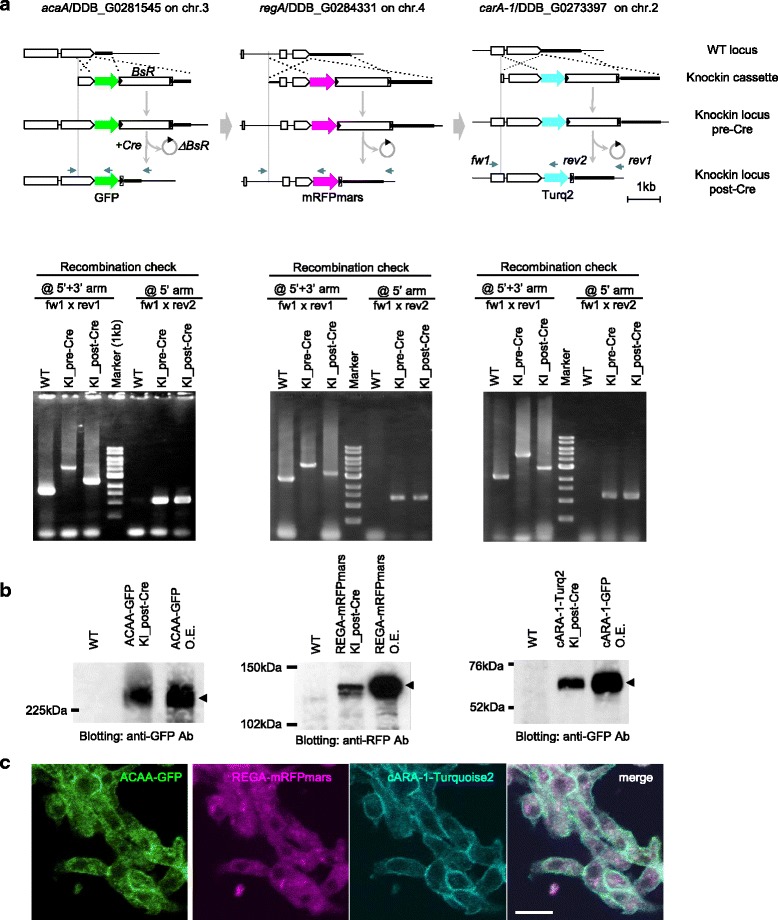
Triple-colour knockin for *ACAA-GFP*, *REGA_mRFPmars* and *cARA-1-Turq2*. **a** Genomic organization of wild-type (WT) and knockin locus (KI) for DDB_G0281545/*acaA* (*left column*), DDB_G0284331/*regA* (*middle column*) and DDB_G0273397/*carA-1* (*right column*) tagged with green, red and cyan fluorescent proteins, respectively. Serial knockin in this order was performed, each followed by *Cre*-mediated *BsR*-recycling. Specific recombination was detected by PCR as in Fig. 2b. Primer fw1/rev1 detects a knockin event (WT to knockin_pre-Cre) and removal of *BsR* cassette (knockin_pre-Cre to post-Cre) as a 2.5 kb increase and 1.7 kb decrease of the amplified band, respectively. The fw1/rev2 set detects specific recombination at the 5′ recombination arm. **b** Protein expression of triple-colour knockin strain. Lysate from over-expressing cells (1/10 volume) with *ACAA-GFP*, *REGA-mRFPmars* and *cARA-1-GFP* were loaded as the positive control. **c** Live confocal images of the triple-colour knockin strain developed for 8 h. *ACAA-GFP* and *cARA-1-Turq2* were localized at the cell surface. *REGA-mRFPmars* was detected in the cytoplasm excluded from the nucleus. Bar: 10 μm

To assess the applicability of the developed strategy, we tried to generate a knockin strain in which multiple genes were labelled with three different coloured fluorescent proteins. For this, we constructed universal knockin modules carrying fusion ready cyan and red fluorescent proteins in addition to *GFP* (Additional file 2: Figure S1B). To overcome the limited availability of the selectable marker in *dicty* cells, *Cre*-mediated recycling of *BsR* gene was utilized [23]. *BsR*-selection marker of *acaA-GFP* strain was removed by the transient expression of *NLS-Cre* (Fig. 3a left column). Restored sensitivity to blasticidin allowed the selection of secondary knockin of *regA-mRFPmars* (Fig. 3a middle column). High knockin efficiency of *regA-mRFPmars* (48 %) was reproduced as in the case for *regA-GFP*. Similarly, removal of *BsR* gene was repeated before and after introduction of *carA-1-Turq2* (Fig. 3a right column). PCR and immunoblotting analysis confirmed successful labelling of *regA* and *carA-1* with red and cyan fluorescent protein, respectively, yielding a triple-colour knockin strain (Fig. 3b). Live imaging of starved cells allowed simultaneous detection of subcellular localization of endogenously expressed *ACAA*, *REGA* and *cARA-1* (Fig. 3c). As expected, *REGA-mRFPmars* was distributed throughout the cytoplasm, except for the nucleus. *cARA-1-Turq2* was evenly presented at the cell membrane [29, 30]. *ACA-GFP* was also detected at the cell periphery like cARA-1, and there were no signs for the polarized accumulation at the rear end of chemotacting cells as reported for over-expressed *ACAA-YFP* [30, 31]. It is not clear the reasons for this, but the different experimental conditions such as the expression level, genetic backgrounds, linkers connecting ACA with FP or imaging conditions might explain mechanisms which should be carefully analyzed in future studies.

## Discussion

We demonstrate here a simple and reliable strategy to establish knockin strains of *Dictyostelium discoidium*, which had been hampered by the difficulty in cloning A/T-rich DNA into circular plasmids. To our knowledge, the pJAZZ system is the unique solution allowing robust handling of unstable DNAs including genomic sequences of *D. discoideum* [17]. As has been recently reported, this enabled development of high quality genomic libraries for the malaria parasite *Plasmodium berghei* (*P. berghei*) and *D. discoideum;* both are characterized by exceptionally high A/T-contents [18, 19]. The unbiased coverage and increased stability with no limitation for the insert size suggest promising applicability for cloning A/T-rich sequences, including promoters and introns of *D. discoideum* which are required for 5′-tagging or promoter replacement.

The linear DNA cloning platform also allows the secondary modification of the cloned insert. As demonstrated here, simple restriction-ligation is feasible to assemble multiple pieces of A/T-rich DNA fragments without any special requisites. Compatibility with other technologies such as recombineering, utilizing *lambda* Red/ET-recombination, and the Gateway system yielded a scalable pipeline that can convert the pJAZZ-based genomic library to thousands of reverse genetic vectors for *P. berghei*)*.* [18]. Its utility has been further strengthened by locus specific barcoding providing a versatile community resource named *Plasmo*GEM [32, 33]. Building the analogous pipeline based on the pJAZZ vector would no doubt accelerate the research activity of *dicty* community.

Another emphasis of our demonstration is in providing practical guidelines in designing a knockin vector for *D. discoideum*. While increasing the length of the homology arm up to 10 kb linearly boosts the recombination efficiency in *P. berghei*)*.* and mice [18, 28], this is not the case for *D. discoideum*. Rather, the critical arm length estimated to be ~3 kb simply controls the knockin event in an almost all-or-none fashion (Table 2). Previously, poor targeting frequency was reported for some loci in which the single cross-over dominated over the double recombination event [34, 35]. *erkB* locus is one such example, as one insertion clone at the 5′ arm was obtained even after extensive screening. In our trial, this was simply overcome by a slightly longer recombination arm, which had not been testable under the constraints of conventional circular plasmid. As the critical length differs depending on the genomic locus, it would be cost and time saving to start with a total of 3 kb of homology arms for the manual construction of reverse genetic vectors.

## Conclusion

The linear DNA cloning system is milestone technology allowing reliable handling of A/T-rich genomic DNAs needed for the reverse genetic analysis of *D. discoideum*. Promoter exchange, site-directed mutagenesis and functional tagging of endogenous proteins will bring new insights into the molecular mechanisms of intercellular communication, self-recognition and kin-discrimination, all of which are involved in social behaviour, one of the most popular research targets of *D. discoideum.* [11, 36, 37].

## Ethics approval and consent to participate

Not applicable.

## Consent for publication

Not applicable.

## Availability of data and material

*pUniv_CKI_mEGFP*, *_Turq2* and *_mRFPmars* (GenBank Accession numbers are KU163138, KU163139 and KU163140, respectively) were deposited in the Dicty stock center.

## Additional files

Additional file 1: Supplementary Methods for Southern blotting. (PDF 767 KB) Additional file 2: Figure S1. Design of universal knockin module. (PDF 905 KB) Additional file 3: Figure S2. Southern blotting analysis for the carA-1 locus. (PDF 2.0 MB)

## Abbreviations

A/T: adenine and thymine
GFP: green fluorescent protein
RFP: red fluorescent protein
cAMP: 3′,5′-cyclic adenosine monophosphate
