# Supplementary material for: Reliable handling of highly A/T-rich genomic DNA for efficient generation of knockin strains of Dictyostelium discoideum
Source: BMC Biotechnol. 2016 Apr 14;16:37. doi: 10.1186/s12896-016-0267-8 (PMC4831088; doi:10.1186/s12896-016-0267-8)
Supplement: Additional file 1: — Supplementary Methods for Southern blotting. (PDF 767 KB) [file 12896_2016_267_MOESM1_ESM.pdf]

Additional file1: Supplementary Methods

Southern blotting

Dictyostelium genomic DNA (*g*DNA) was purified as described elsewhere (Dictybase; http://dictybase.org/techniques/molec_biol/genomicDNA_1.html) and was digested with XbaI for 18 hours. 20 μg of digested *g*DNA (1.5×10^6 cell/Lane) was separated by 0.7 % agarose gel electrophoresis and was transferred onto nylon membrane (Roche). Preparation, hybridization and detection of DIG-labelled DNA probes was performed by using DIG-High Prime DNA Labeling and Detection Kit II (Sigma-Aldrich) according to the manufacturer's instructions. Briefly, the blots were hybridized with DIG-labelled DNA probes (Fig. S2A) at 42 °C for 18 hours, then washed twice with 0.1 % SDS and 0.5 × SSC at 65 °C for 15 minutes, incubated with Alkaline Phosphatase-conjugated anti DIG antibody (kit supplied, sheep, 15,000 × diluted) at 23 °C for 15 minutes. Chemi-luminescent signals of CSPD reaction were detected with X-ray film (Fuji) for 45 minutes.
