# Supplementary material for: Reliable handling of highly A/T-rich genomic DNA for efficient generation of knockin strains of Dictyostelium discoideum
Source: BMC Biotechnol. 2016 Apr 14;16:37. doi: 10.1186/s12896-016-0267-8 (PMC4831088; doi:10.1186/s12896-016-0267-8)
Supplement: Additional file 2: Figure S1. — Design of universal knockin module. (PDF 905 KB) [file 12896_2016_267_MOESM2_ESM.pdf]

A

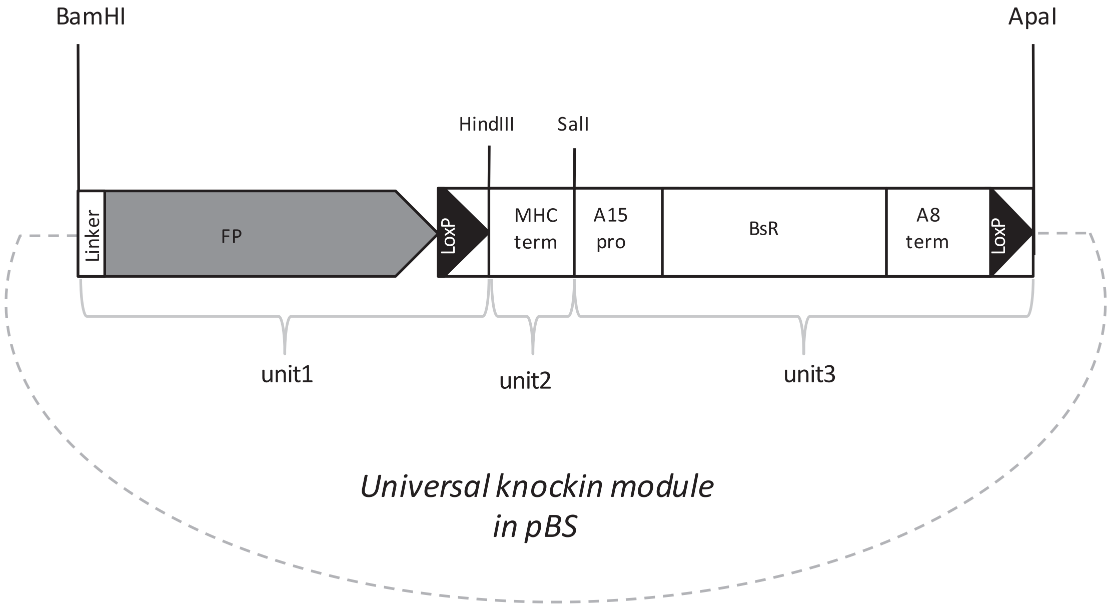

B

Plasmid Name	*FP*	*Codon for FP*	*Fusion Linker (in amino acid code)*
pUniv_CKI_mEGFP	*mEGFP*	*A.*	-GS-GAPSGGGATAGAGGAGGPAGRSGG-Met of FP
pUniv_CKI_Turq2	*Turquoise2*	*A.v*	-GS-GAPSGGGATAGAGGAGGPAGRSGG-Met of FP
pUniv_CKI_mRFPmars	*mRFPmars*	*D.d*	-GS-GAPSGGGATAGAGGAGGPAGRSGG-Met of FP

Additional file2: Figure S1. Design of universal knockin module.

A) Schematic illustration of the universal knockin module cloned into pBluescript. FP with linker; cDNA of fusion ready fluorescent protein, MHC term; terminator of myosin heavy chain A, A15 pro; actin15 promoter, BsR; blasticidin resistance gene, A8 term; actin8 terminator.

B) Summary of three plasmids encoding fusion ready green, cyan and red fluorescent proteins. CKI; C-terminal knockin, *A.v; Aequorea victoria, D.d; Dictyostelium discoideum*, fusion linker indicates the peptide sequence connecting C-terminal end of GOI with fluorescent proteins.
