# Supplementary material for: Reliable handling of highly A/T-rich genomic DNA for efficient generation of knockin strains of Dictyostelium discoideum
Source: BMC Biotechnol. 2016 Apr 14;16:37. doi: 10.1186/s12896-016-0267-8 (PMC4831088; doi:10.1186/s12896-016-0267-8)
Supplement: Additional file 3: Figure S2. — Southern blotting analysis for the carA-1 locus. (PDF 2.0 MB) [file 12896_2016_267_MOESM3_ESM.pdf]

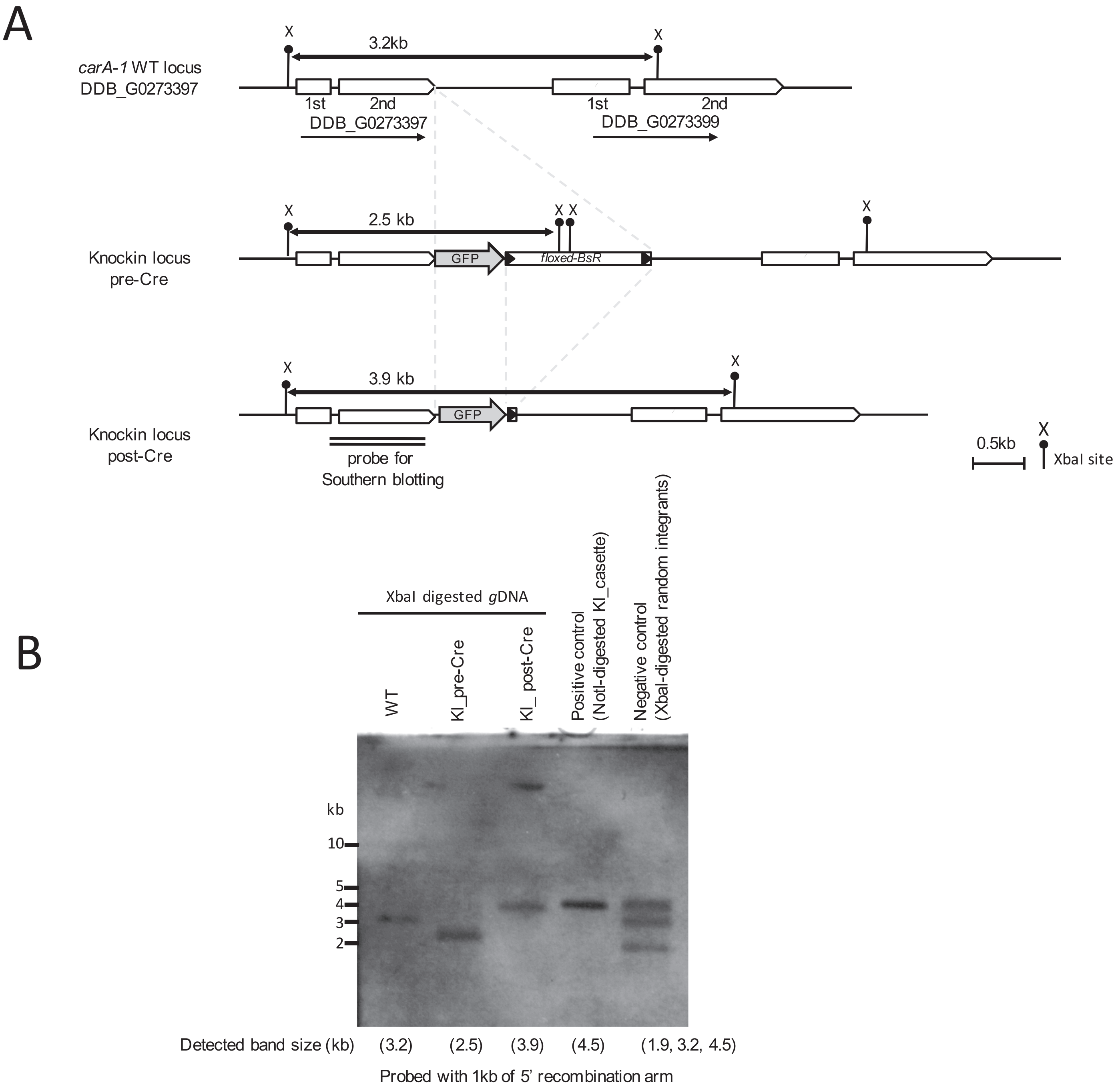

Additional file3: Figure S2. Southern blotting analysis for the *carA-1* locus.

A) The genomic organization of DDB_G0273397/*carA-1* with its downstream gene, DDB_G0273399/*mip-1*. X indicates cutting sites for XbaI. The double line represents the probe position for Southern blotting (1 kb of the terminal exon of carA-1, corresponding to the 5' recombination arm of the knockin vector). Modified from Fig. 2A.

B) DNA gel blots of wildtype (WT) and knockin cells (KI) for DDB_G0273397/*carA-1*. Changes in the size of detected bands from 3.2 kb (WT) to 2.5 kb (KI_pre_Cre) and 3.9 kb (KI_post_Cre) indicates the specific knockin homologous recombination and the removal of the *floxed-BsR* cassette, respectively. NotI digested knockin cassette and the genomic DNA of random integrants (at least 2 integrations). The position of DNA size markers is indicated on the left.
